# Autumn temperatures at African wintering grounds affect body condition of two passerine species during spring migration

**DOI:** 10.1371/journal.pone.0217619

**Published:** 2019-05-29

**Authors:** Irith Aloni, Shai Markman, Yaron Ziv

**Affiliations:** 1 Spatial Ecology Laboratory, Department of Life Sciences, Ben-Gurion University of the Negev, Beer Sheva, Israel; 2 Department of Biology & Environment, Faculty of Natural Sciences, University of Haifa—Oranim, Israel; University of Pavia, ITALY

## Abstract

Most papers on the physical condition of birds during spring migration focused on food availability preceding migratory take-off. Only a few studies examined the effect of climate conditions at the wintering grounds upon autumn arrival on bird physical condition later on. Here, we hypothesized that environmental conditions upon arrival at the wintering grounds, and not necessarily upon departure, have a crucial carry-over effect on bird spring migration. Using 29,000 observations of the lesser whitethroat, *Sylvia curruca*, and the eastern Bonelli’s warbler, *Phylloscopus orientalis*, we found temperatures upon arrival at the African wintering grounds to be the only climatic variable correlated with birds’ body state upon spring stopover in Israel, six months later. Two different mechanisms could explain these results. One possibility is that high temperatures create favorable conditions for insect activity, which allows rapid recovery from autumn migration and hence successful winter survival and maintenance. Another possible scenario is that harsh conditions, due to the heat and dry environment, cause high mortality, permitting survival of larger individuals which, then, enjoy reduced inter- and intra-specific competition. Whatever the mechanism is, our findings suggest that conditions upon autumn arrival, and not necessarily at the end of winter as traditionally thought, may have a major impact on migrating birds.

## Introduction

In an extensive review of the reasons for recent declines in migratory bird populations, Vickery *et al*. [1] point out habitat loss and degradation and climate change as the most influential factors. Many studies in recent years have focused on the effect of changing climate on bird migration and survival [1–8]. Migrating birds, particularly long-distance migrants, are more susceptible to environmental changes than their resident counterparts due to their complicated life cycle, which relies on various habitats and locations at different times along the year [1,9–10]. Food availability at wintering grounds has been pointed out by several researchers as a key factor affecting the birds’ physical condition and their ability to complete spring migration successfully and timely [3,6,9,11–17]. Additionally, many studies suggest that favorable conditions during winter induce advanced migration in spring, which potentially increases the chances to breed successfully [2–3,11,17–20]. Moreau [21] pointed out that numerous Afro-Palaearctic migrants wintering in the Sahel experience deteriorating ecological conditions along their stay due to the semiarid climate. The rainy season in the eastern Sahel takes place in summer, just before the arrival of the migrating birds, and the area dries up during the birds’ overwintering period. Moreau emphasized the difficulty that birds face as they have to prepare for spring migration when conditions are worst. This phenomenon is known as the Moreau’s Paradox [11].

However, very few studies have directly examined possible effects of environmental conditions at wintering grounds on bird physical condition during migration [3,5,11]. In a study of the American redstart, *Setophaga ruticilla*, Studds and Marra [3,20] revealed that rainfall in the Jamaican wintering grounds strongly affected food availability, body mass, and spring departure schedules of the birds. Schlaich *et al*. [11], who tracked 36 Montagu’s Harriers, *Circus pygargus*, during their wintering time in Senegal, found that the birds responded to the decreasing food availability by increasing their flight time during the second half of the winter. Additionally, birds departed later on springs with lower Normalized Difference Vegetation Index values and presumably lower food abundance and consequently also arrived later at their breeding site. Smith *et al*. [15], in a study of the northern waterthrush, *Seiurus noveboracensis*, in its tropical non-breeding grounds, found that fat deposition preceding spring migration was faster among birds occupying wetter habitats. González-Prieto and Hobson [16] examined nutritional conditions and arrival phenology of several Nearctic-Neotropical species, and found that birds arrived later and in a lower nutritional condition during colder springs.

Food availability at wintering grounds may also affect the moult of those species which undergo moult during the wintering season [2,22]. Van den Brick *et al*. [22] showed that rainfall in Botswana affected moult rate in the barn swallow, *Hirundo rustica*. Moult rate as well as food availability affected feather quality, which in turn affected flight performance and thermoregulation capacity [2]. For those species that moult immediately upon arrival at their wintering grounds, environmental conditions at this time may be crucial.

One possibility that has never been considered in the literature is that climate conditions and food availability upon arrival at the wintering grounds in autumn may affect birds’ physical condition throughout winter and even during the subsequent spring. Clearly, conditions upon autumn arrival affect bird survival [1]. Long-distance migrants arriving at wintering grounds are exhausted after their long migratory flight [23], which includes major obstacles such as the Mediterranean Sea and/or the Sahara Desert. Additionally, many birds have just completed a demanding breeding season, or, in the case of juveniles, their very first migratory experience. It is, therefore, likely that conditions upon arrival at wintering grounds would be crucial for survival. Moreover, in the case of species which winter along the African semi-arid grassland, where winter is mostly dry, environmental conditions upon arrival may have a carry-over effect in terms of the birds’ physical condition which may affect migration in the following season.

In this study, we focused on birds wintering in the east African semi-arid grassland and scrubland to address the following question: Is there a carry-over effect of climate conditions at the wintering grounds of long-distance migrants on the birds’ physical condition during spring migration? We tested our novel hypothesis that climate conditions upon arrival at wintering grounds are crucial for spring migration body state against the more traditional hypothesis that climate conditions preceding spring departure are most important. These two hypotheses are not necessarily mutually exclusive, and it is quite possible that both, conditions in autumn and in early spring, influence the birds’ body state during spring migration.

## Methods

We obtained all bird data from the International Birding and Research Centre in Eilat (IBRCE), Israel. The IBRCE is managed by the Israel Nature and Parks Authority, the very same authority that provides bird ringing licenses. Therefore, all people who ring birds at the IBRCE must hold a valid ringing license. The data we used was all from regular ringing procedures, in which birds are captured, ringed, measured, and immediately released. There is no requirement for an ethic committee approval for regular bird ringing.

In order to test our hypotheses, we studied the body weight and body state (body weight to wing length ratio) of two passerine species, the lesser whitethroat, *Sylvia curruca*, and the eastern Bonelli's warbler, *Phylloscopus orientalis*, upon their spring arrival in Eilat, Israel. Israel is located on a migratory bottleneck through which about half a billion birds pass twice a year [24]. Eilat, located at the very southern tip of Israel, is a major stopover oasis, right after crossing the Sahara Desert [25]. The two species chosen for analysis arrive in Eilat in large numbers and have a winter distribution that is limited mostly to the semi-arid zone of North Africa, south of the Sahara desert belt [26–27]. Both species are mostly insectivorous.

The dataset of bird spring arrivals (February to May) included 26,034 individuals of *S*. *curruca* trapped during 1984–2013 and 2,768 individuals of *P*. *orientalis* from 1983–2013 (S1 Appendix). Ringing activity at the IBRCE takes place along the spring season, between mid-February and mid-June. Mist nets are set up daily for the first four hours of day light. All caught birds are ringed if not yet ringed, measured (weight, wing length, and fat score), and released. Weight is measured with Electronic scales to an accuracy of 0.1 g. Wing length is measured following Svensson [28], from the carpal joint to the tip of the longest primary on the closed wing, while the wing is flattened and straightened. Measurements are to an accuracy of 1 mm.

For the wintering grounds of each species, we followed the species distribution maps available at the Birdlife International website [26–27]. The wintering grounds of *P*. *orientalis* are located in eastern Africa, and the entire range was included in the analysis. The distribution range of *S*. *curruca* is much wider in both winter and summer quarters, and includes large regions in Asia. According to Shirihai *et al*. [29] and Kiat *et al*. [30], most European populations of *S*. *curruca* migrate back north through the Eastern Mediterranean Flyway. Hence, we included all of the species African wintering range (in North-Eastern Africa) but the isolated population range in Mali. Additionally, we included the south-western portion of the Arabian Peninsula, as it seemed possible that birds would fly from there along the Red Sea to Eilat. For this portion, we included the area lying west of the diagonal connecting N 15^0^ E 49^0^ and N 25^0^ E 42^0^, and the area west of the straight line connecting N 25^0^ E 42^0^ and N 28^0^ E 42^0^.

Information regarding arrival time of the birds at the wintering grounds is scarce. *S*. *curruca*’s autumn migration through Israel takes place between late July and early November. Most birds’ passage occurs in August, which suggests that birds arrive at their wintering grounds in late August or September. However, some birds, particularly juveniles, may linger until November [25,31]. According to Bairlein *et al*. [32], the birds start arriving in Sudan in August but the passage to Chad occurs mostly in October. *P*. *Orientalis* passes through Israel between mid-July and the end of September [25] and through Egypt during August and September [33]. Spring arrivals of both species in Eilat begin in late February, but most birds arrive between March and May. Given this information, where many birds arrive in Africa in September, and mostly start leaving not before March, we chose to use the time span between September and February for our analysis.

Climate data at the wintering grounds were downloaded from the Climate Research Unit website of the University of East Anglia, UK [34]. We applied the species winter distribution maps to the climate datasets to extract the relevant region for each species separately. Climate data used included temperature and precipitation variables along the wintering season, variables which are most influential in terms of vegetation growth and insect activity and, thus, food abundance [2–3,12–13,29,35]. Overall, 28 different variables have been used for the analyses. For temperature, we used monthly maximal, minimal and daily means for each month of the wintering season—September to February. For precipitation, particularly in rather dry environments such as the wintering grounds analyzed here, annual quantities may be more crucial than monthly amounts, especially in light of the fact that the rainy season takes place during summer, before the birds arrive. Hence, in addition to mean monthly precipitation levels for each of the wintering months, we included the following variables: (1) Annual precipitation for the year preceding spring departure (from March of the previous year to the current February); (2) June to August accumulated precipitation (summer precipitation); (3) September to November accumulated precipitation (autumn precipitation); (4) December to February accumulated precipitation (winter precipitation).

**Fig 1:**
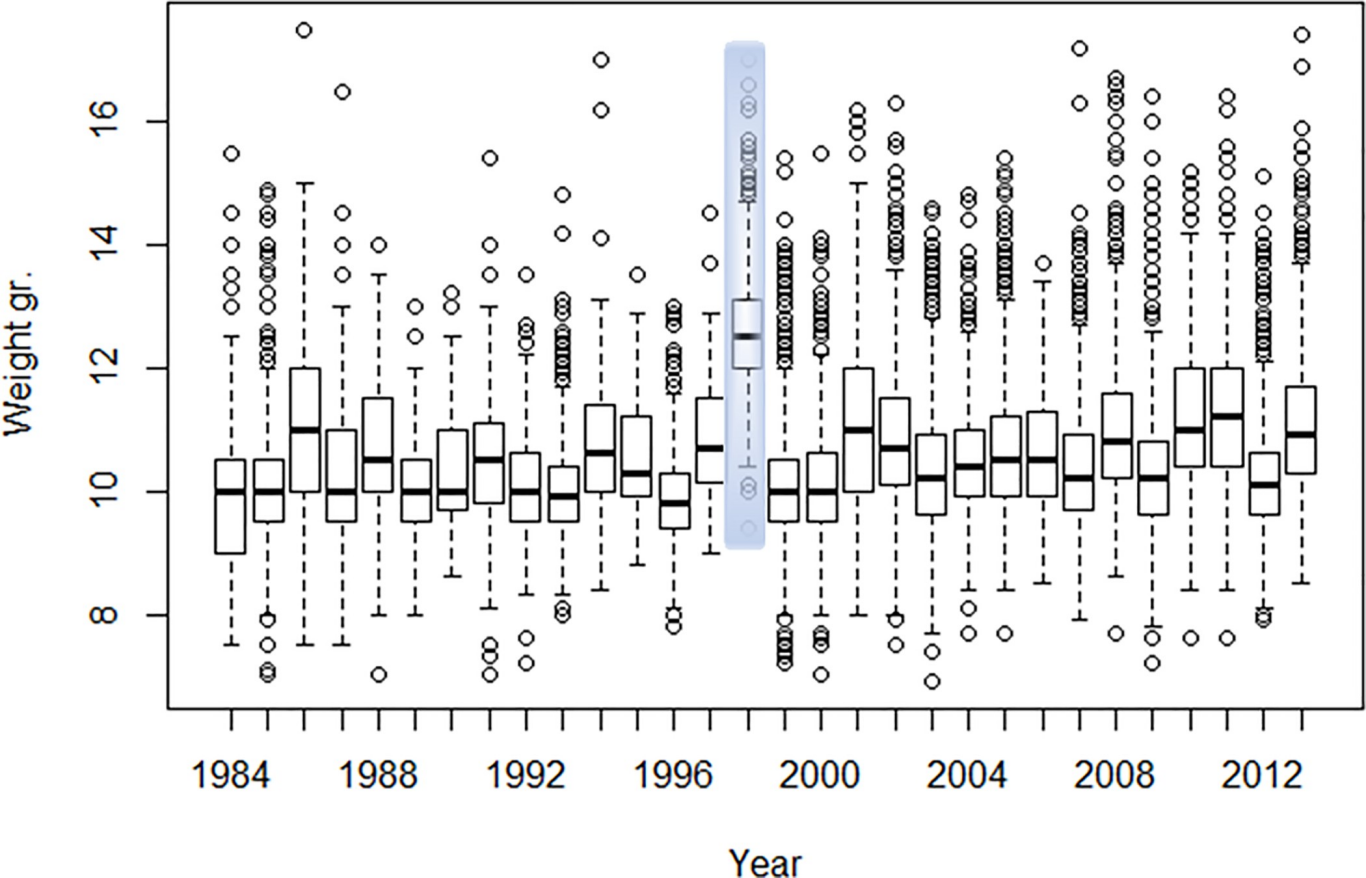
Boxplots of weight upon spring arrival in Eilat, Israel, of *Sylvia curruca*. 1998 weight range, marked in shaded gray, was excluded from the analysis. The central box represents the central 50% of observations for each year, where the central line of the box represents the median, and the box lower and upper edges represent the first and third quartiles respectively. The whiskers represent the length of 1.5 times the interquartile range (IQR).

**Fig 2:**
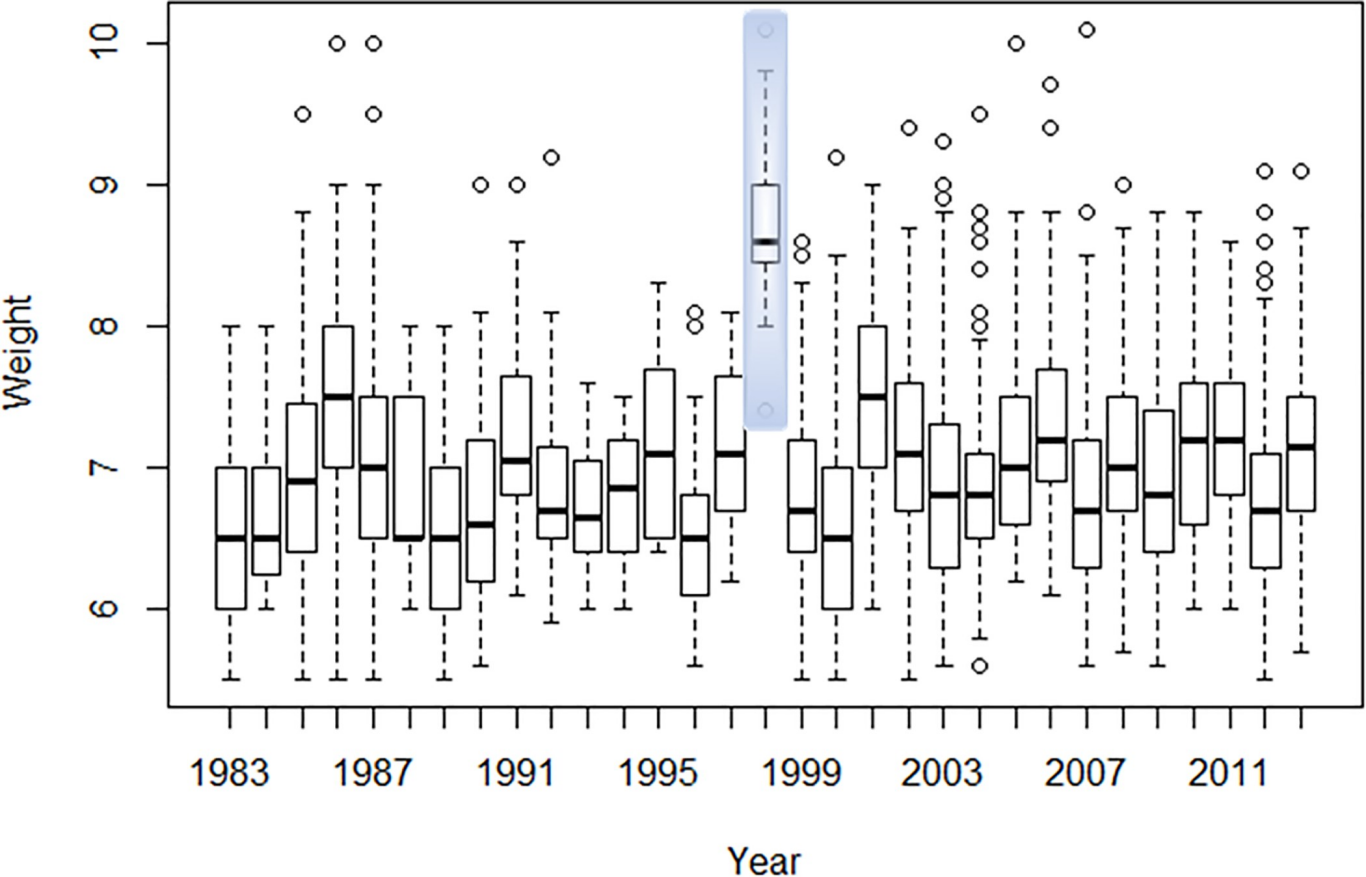
Boxplots of weight upon spring arrival in Eilat, Israel, of *Phylloscopus orientalis*. 1998 weight range, marked in shaded gray, was excluded from the analysis. The central box represents the central 50% of observations for each year, where the central line of the box represents the median, and the box lower and upper edges represent the first and third quartiles respectively. The whiskers represent the length of 1.5 times the interquartile range (IQR).

Mean body weight and mean body state of each species upon spring arrival in Eilat were calculated for each year. Body state (weight to wing length ratio, sometimes referred to as body condition) is a measure of body condition which supposedly controls for phenotypic size differences between individuals [36]. Examination of the weight distribution along the years revealed exceptionally high values in 1998. The entire range of observations in that year was rather off the range of all other years for both species, with less than the highest 20% of all years’ data overlapping with 1998 data (Figs 1 & 2). As a result, the mean weights of 1998 fell far beyond the samples upper 99% confidence interval. This raised questions as for possible technical problem on that particular year, and, hence, 1998 was omitted from the analyses (which eventually lead to more conservative results). Yearly mean weight and body state were then correlated with the temperature and precipitation variables of the wintering grounds using Pearson’s correlation coefficient.

Then, a principal component analysis (PCA) was run on the entire set of 28 temperature and precipitation variables in order to get a smaller, perpendicular set of variables for a formal analysis. However, high correlations between monthly temperatures along the season (Pearson’s r of up to 0.65 among different months’ temperatures) resulted in PC1 which incorporated about 70–80% for each of most of the temperature variables. This result would mask different effects of temperature on the birds at different times of the year. Therefore, we assembled a set of 5 variables, out of the 28 original ones, which contain the major information of the original set while keeping the distinction between autumn and winter components. Each of these new variables represents one season, summer, autumn or winter (rather than one month), and each refers either to temperature or precipitation. For precipitation we simply used the accumulated precipitation within the specified season. For temperature we chose the average maximal temperature of the specified season, as we assumed that it may have the most important effect on food abundance through an effect on insect activity (direct and indirect). Since temperatures in this area rarely get cold enough to affect bird body temperature in any significant way, minimum temperatures seemed less relevant. Thus, the new set of variables included: (1) Autumn mean maximum temperature (average of mean monthly maximal temperature for September to November); (2) Autumn precipitation (accumulated mean precipitation for September to November); (3) Winter mean maximum temperature (average of mean monthly maximal temperature for December to February); (4) Winter precipitation (accumulated mean precipitation for December to February); (5) Summer precipitation (accumulated mean precipitation for June to August).

These new variables were mostly uncorrelated, except for a correlation between autumn and winter temperatures for both species, and between summer and autumn precipitation for the lesser whitethroat only. In particular, there was no correlation between any of the temperature and precipitation variables. These five variables were than used in order to create a model of body state as a function of African climate conditions for each species. Forward, backward, and bidirectional stepwise regressions, starting from a null, full no interactions, full 2-way interactions, and full 3-way interactions models were applied to the data sets of each species. For basic models with a single explanatory variable, a cut off level of 0.05 was allowed. Machine Akaike Information Criteria (AIC) as well as manual F-test criteria (using p-value cutoff of 0.01) were used for further factor addition/elimination [37]. All analyses were conducted using the R software version 3.3.1 [38].

The original dataset included approximately 29,000 observations. However, due to the use of means of bird parameters as response variables in the analyses, the size of the dataset is not explicitly expressed. The degrees of freedom are based on the number of years included in analysis, not the number of birds. Yet, the mean body state and body weight values, particularly those of *S*. *curruca*, are most representative of the real population, due to the very large size of the bird datasets. This ensures that the averages are not biased by some exceptional outliers.

## Results

Correlation analysis of climate conditions at the African wintering grounds and bird body state and weight upon spring arrival in Eilat, Israel, demonstrated a similar pattern for both *S*. *curruca* and *P*. *orientalis*. We found statistically significant and positive correlations at the 0.01 and 0.05 levels between autumn temperatures at the wintering grounds and both bird body state and weight (Table 1). For *S*. *curruca*, correlations at these levels were observed for September, October and November, whereas for *P*. *orientalis* we found such correlations only in October. Maximal daily temperature showed the highest correlations in most cases. Neither of the temperature variables during later months, nor any of the precipitation variables showed any significant correlation with body state or weight (Tables 1 & 2).

**Table 1:** Pearson’s correlation coefficients between temperatures at African wintering grounds and mean body weight and body state (weight to wing length ratio) upon spring stopover in Eilat, Israel, of two long-distance migratory passerines.

	*Sylvia curruca*		*Phylloscopus orientalis*	
Variable	Mean weight	Mean body state	Mean weight	Mean body state
**Sep.**				
Mean Temp.	0.38*	0.32	0.21	0.17
Mean Min. Temp.	0.29	0.23	0.17	0.10
Mean Max. Temp.	0.42*	0.37	0.23	0.20
**Oct.**				
Mean Temp.	0.49**	0.44*	0.43*	0.41*
Mean Min. Temp.	0.48*	0.42*	0.35	0.34
Mean Max. Temp.	0.48*	0.43*	0.47*	0.43*
**Nov.**				
Mean Temp.	0.44*	0.43*	0.28	0.28
Mean Min. Temp.	0.42*	0.40*	0.25	0.27
Mean Max. Temp.	0.45*	0.44*	0.30	0.29
**Dec.**				
Mean Temp.	0.29	0.29	0.30	0.29
Mean Min. Temp.	0.21	0.21	0.31	0.30
Mean Max. Temp.	0.33	0.34	0.28	0.28
**Jan.**				
Mean Temp.	0.21	0.20	0.31	0.26
Mean Min. Temp.	0.17	0.16	0.26	0.21
Mean Max. Temp.	0.23	0.23	0.34	0.29
**Feb.**				
Mean Temp.	0.24	0.23	0.29	0.23
Mean Min. Temp.	0.26	0.25	0.29	0.24
Mean Max. Temp.	0.22	0.21	0.28	0.22

* Significant at α = 0.05

** Significant at α = 0.01

**Table 2:** Pearson’s correlation coefficients between precipitation at African wintering grounds and mean body weight and body state (weight to wing length ratio) upon spring stopover in Eilat, Israel, of two long-distance migratory passerines.

	*Sylvia curruca*		*Phylloscopus orientalis*	
Variable	Mean Weight	Mean body state	Mean Weight	Mean body state
Pre.^1^ September	-0.13	-0.08	-0.11	-0.10
Pre. October	0.03	0.11	-0.25	-0.14
Pre. November	0.22	0.23	0.06	0.07
Pre. December	0.01	0.03	0.24	0.21
Pre. January	-0.16	-0.14	-0.09	-0.06
Pre. February	0.05	0.02	-0.02	-0.02
Annual mean Pre. (Mar.-Feb.)	0.17	0.18	-0.09	-0.07
Summer Pre. (Jun.-Aug.)	0.20	0.19	-0.04	-0.03
Autumn Pre. (Sep.-Nov.)	-0.02	0.05	-0.21	-0.14
Winter Pre. (Dec.-Feb.)	-0.03	-0.03	0.14	0.13

^1^Pre—precipitation

**Fig 3:**
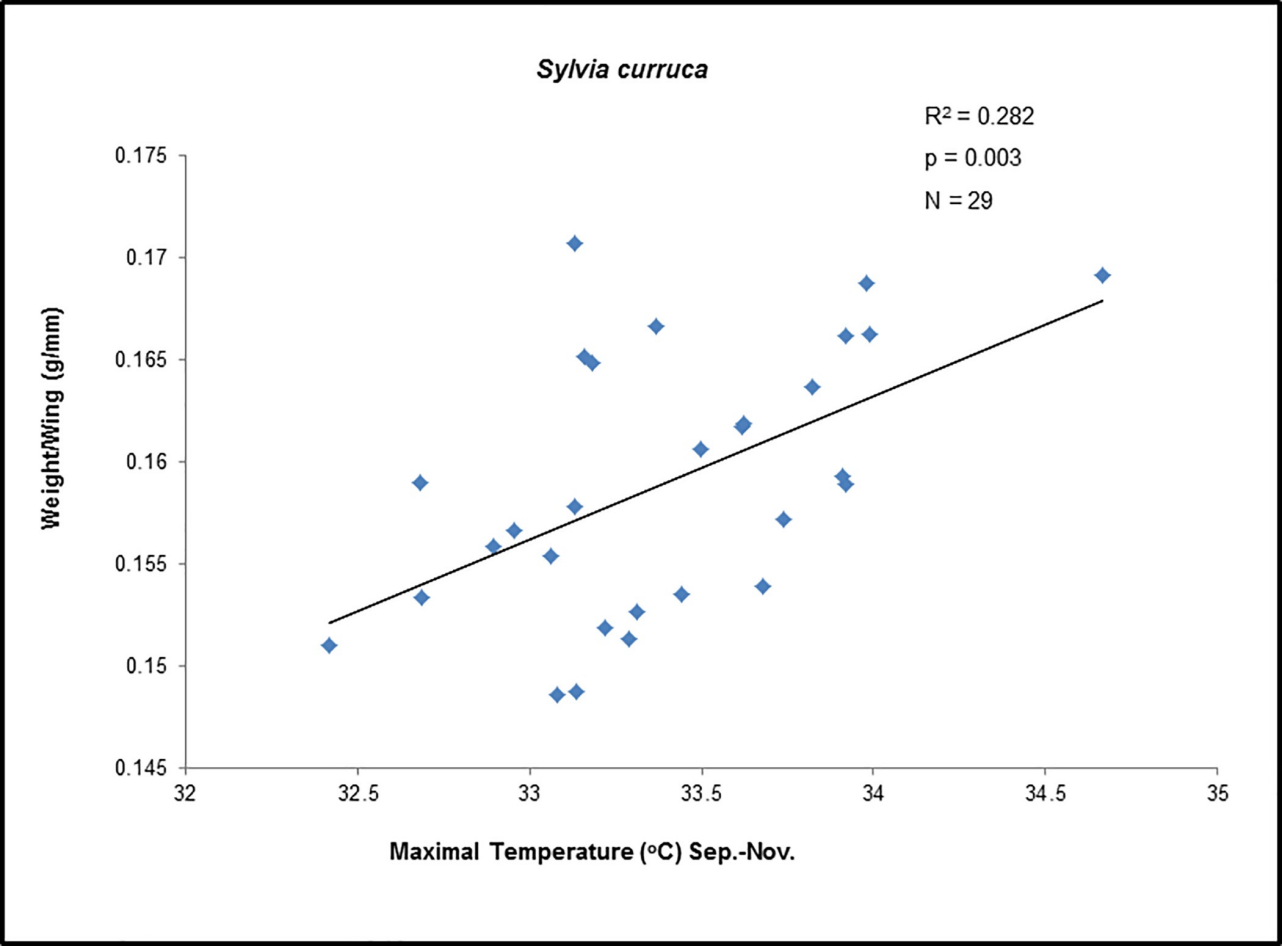
Mean yearly body state of the lesser whitethroat, *Sylvia curruca*, upon spring arrival in Eilat, Israel, as a function of autumn maximum temperature at African wintering grounds, 1984–2013 (1998 excluded).

**Fig 4:**
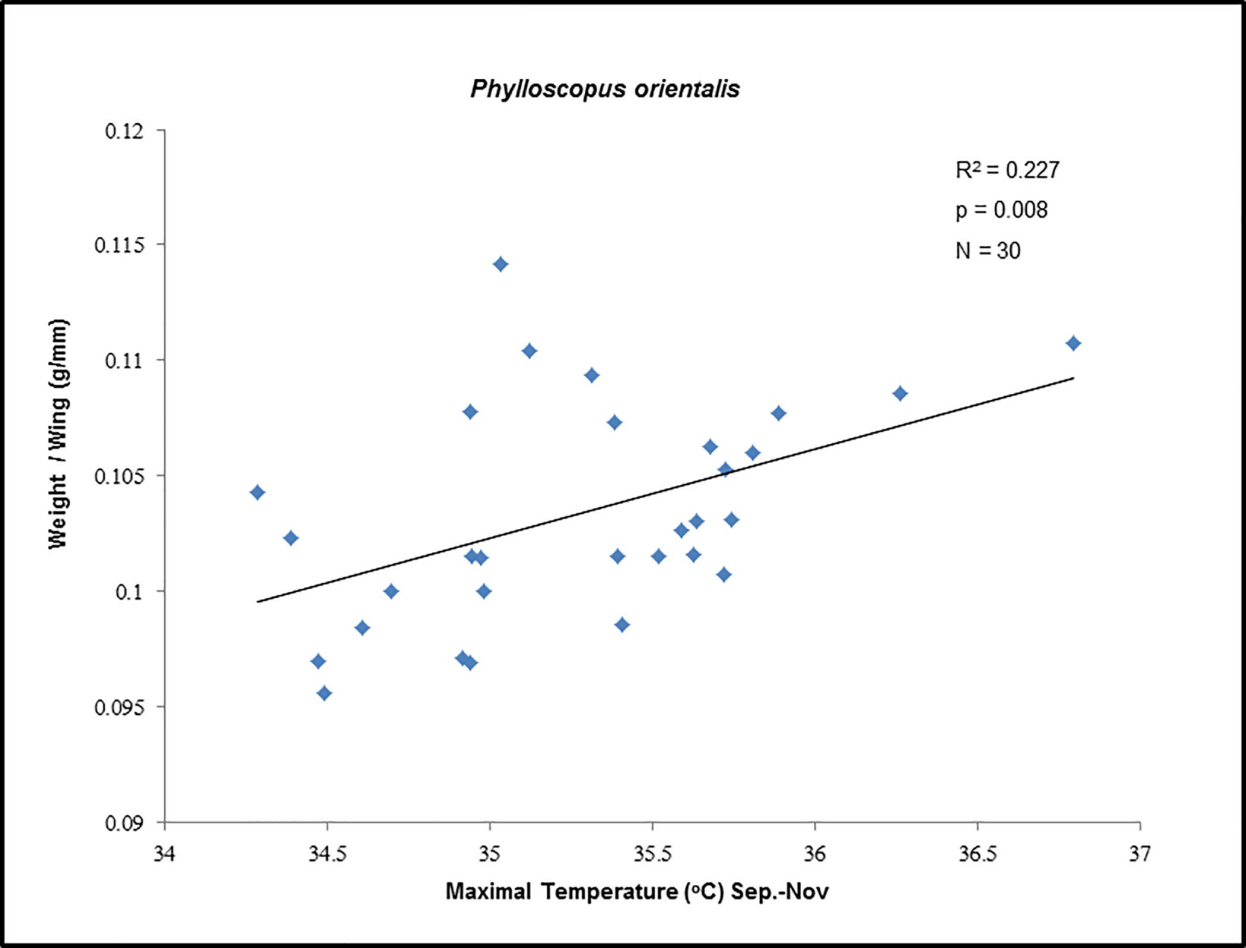
Mean yearly body state of the eastern Bonelli’s warbler, *Phylloscopus orientalis*, upon spring arrival in Eilat, Israel, as a function of autumn maximum temperature at African wintering grounds, 1983–2013 (1998 excluded).

For both *S*. *curruca* and *P*. *orientalis*, the multiple regression analyses resulted all in the simplest model, with body state being a function of autumn mean maximum temperature (Table 3). None of the other variables used for the analysis showed any relationship with mean body state, neither as a simple model component nor within an interaction term. The various stepwise regressions have all led to this same basic model, no matter what the initial model used was. Both models were statistically significant with a p-value of 0.003 for *S*. *curruca* (Table 3A), and of 0.008 for *P*. *orientalis* (Table 3B). The effect of autumn maximum temperature on bird body state was positive in both species, indicating an increase in body state with increasing autumn maximum temperatures (Figs 3 & 4).

**Table 3:** Final models for body state (weight to wing length ratio) as a function of climate variables, 1983–2013, excluding 1998 (see Methods).

**A. *Sylvia curruca***							
Body State ~ MxTmp.SN*							
	Coefficients:						
			**Estimate**	**Std. Error**	**t-statistic**	**Pr(>|t|)**	
	(Intercept)		-0.0753	0.072	-1.045	0.3054	
	MxTmp.SN		0.007	0.0022	3.252	0.0031	**
	**R**^**2**^ **= 0.282**		**Adjusted R**^**2**^ **= 0.255**				
	**F**_**(1,27)**_ **= 10.58**		**p-value = 0.003**				
**B. *Phylloscopus orientalis***							
Body State ~ MxTmp.SN*							
	Coefficients:						
			**Estimate**	**Std. Error**	**t-statistic**	**Pr(>|t|)**	
	(Intercept)		-0.0326	0.0474	-0.6889	0.4966	
	MxTmp.SN		0.0039	0.0013	2.871	0.0077	**
	**R**^**2**^ **= 0.227**		**Adjusted R**^**2**^ **= 0.200**				
	**F**_**(1,28)**_ **= 8.24**		**p-value = 0.0077**				

*MxTmp.SN–Mean maximal temperature during September to November

## Discussion

For both the lesser whitethroat and the eastern Bonelli’s warbler, we found a highly significant positive relationship between autumn temperatures at the wintering grounds and bird body state and weight upon spring arrival in Eilat, Israel. These findings strongly support our carry-over hypothesis stating that environmental conditions upon autumn arrival at the wintering grounds can have a crucial impact on bird physical condition throughout the wintering season and into spring migration. The correlations between November temperatures and body state and weight for the lesser whitethroat (Table 1) further support this notion, as autumn migration of this species lingers into November [25]. This idea of carry-over effect of autumn temperatures is further sustained by the absence of any correlations between either temperatures of later months or any of the precipitation variables with body state or weight for any of the species (Tables 1 & 2). These results contradict the traditional presumption that conditions immediately preceding departure from wintering grounds are of upmost importance [1,3,13,15], at least for these two species.

The few studies at African wintering or staging grounds that examined correlations of bird survival and spring arrival time with environmental conditions were conducted mostly in Western Africa. In that region, severe droughts resulting in the drying of the Sahel’s seasonal water bodies, led to a catastrophic food shortage and a major decline in bird numbers [1,6,10,12–13]. Our study has focused on species which spend their winter mostly in eastern Africa, in a semi-arid habitat. The major rainy season in this region is during summer (June to September), while the winter is dry [39]. Annual precipitation amounts to a few hundred millimeters (250–400 mm for *S*. *curruca*, 200–450 mm for *P*. *orientalis*). Hence, these species must be acclimated to wintering under relatively arid conditions, which may explain the lack of correlations with precipitation variables.

In this study, we have not only examined the climate conditions preceding spring departure, but also the conditions prevailing upon autumn arrival at the wintering grounds. Since the rainy season at the wintering grounds takes place during summer, food availability upon autumn arrival should be at its peak. High temperatures, particularly when combined with wet conditions (which prevail during summer and fall in this region), accelerate insect developmental rates and activity [13,25,35]. Higher insect activity should reduce search time, energy expenditure, and competition and, therefore, enhance bird recovery and improvement of body mass and condition.

The birds arrive exhausted from a long and demanding migratory trip, which consists of major ecological obstacles including the Mediterranean Sea and/or the Sahara Desert. Moreover, just before autumn migration, many of the birds undergo a demanding breeding season, and adults of the lesser whitethroat also undertake a complete moult. For juveniles, this is their first migratory experience. Therefore, favorable conditions upon arrival at the wintering grounds may be of crucial importance, as they would facilitate a rapid and complete recovery [40]. A good body condition on the verge of the dry season would affect foraging efficiency and, thus, enable a successful passage of winter and a timely and effective preparation for spring migration, which may be expressed in body state upon arrival in Eilat.

There is an alternative scenario that can explain the observed high correlations between autumn temperatures at the wintering grounds and bird body state upon spring arrival in Eilat, Israel. It is possible that high autumn temperatures imply a rapid dry up after the summer rains. Shortage of food as of a hot and dry autumn may lead to a high mortality rate of weak birds, while selecting for the survival of larger and stronger ones. As a result, the surviving birds shall enjoy a reduced competition pressure, both inter- and intra-specific, which may allow more resources for the winter and for the pre-migratory fat assimilation. Thus, the hot autumn shall bring about larger birds in Eilat in spring in two ways: selection would lead to the survival of larger birds, and the resulting reduced competition may allow a higher body mass gain.

Some support for our proposition that conditions during autumn exert a long-lasting carry-over effect on the birds’ physiological condition in spring and even in the following summer is provided by Schaub *et al*. [41]. Schaub and his colleagues examined the effect of environmental variation at the breeding, staging and wintering grounds of the red-backed shrike, *Lanius collurio*, on its survival rate, spring arrival phenology, and reproductive success. They found that the red-backed shrike produced more fledglings on years following higher primary productivity in the Sahel autumn passage region. Moreover, similarly to our results in the current study, Schaub *et al*. [41] did not find any significant impact of conditions later in winter or early spring. Goodenough *et al*. [42] found that female breeding success of the Pied Flycatchers, *Ficedula hypoleuca*, was related to isotopic signature of feathers grown in Africa during winter, and suggested that this may reflect a long-lasting carry-over effect of wintering habitat on summer breeding performance. Some more support to the carry-over effect comes from a study by Sicurella *et al*. [43], who studied population dynamics of the barn swallow. Their results indicated that primary productivity at the wintering grounds upon autumn arrival influenced population dynamics more than habitat conditions at the breeding grounds.

Ockendon *et al*. [6] refer to precipitation during summer in a similar habitat (and latitudes) in Western Africa, stating that it may be critical in determining overwinter survival and bird condition upon migration later on. Indeed, they found high correlations between precipitation at the wintering grounds and bird numbers in the following summer in England. Interestingly, they found no correlation for *S*. *curruca* with precipitation, which agrees with our findings.

To the best of our knowledge, our study is the first to examine the effect of climate conditions at wintering grounds upon autumn arrival and during the wintering season on bird body state and weight during spring stopover on the Afro-Palaearctic migration route. Our results highlight the importance of environmental conditions upon autumn arrival at the African wintering grounds for long-distance migratory birds. We claim that conditions upon autumn arrival are crucial for the birds' physical state not only upon arrival, but months later, during spring migration. We suggest that it is important to further investigate the role of environmental conditions upon autumn arrival at African wintering grounds in generating long-lasting impacts on bird body state as well as long-term survival and reproductive success.

## Supporting information

S1 Appendix Bird data. (XLSX)
